# Demonstration of Thin Film Bulk Acoustic Resonator Based on AlN/AlScN Composite Film with a Feasible $K_{eff}^{2}$

**DOI:** 10.3390/mi13122044

**Published:** 2022-11-22

**Authors:** Laixia Nian, Yang Zou, Chao Gao, Yu Zhou, Yuchen Fan, Jian Wang, Wenjuan Liu, Yan Liu, Jeffrey Bowoon Soon, Yao Cai, Chengliang Sun

**Affiliations:** 1 The Institute of Technological Sciences, Wuhan University, Wuhan 430072, China; 2 School of Microelectronics, Wuhan University, Wuhan 430072, China

**Keywords:** film bulk acoustic resonator, aluminum scandium nitride, composite film, effective electromechanical coupling coefficient

## Abstract

Film bulk acoustic resonators (FBARs) with a desired effective electromechanical coupling coefficient ($K_{eff}^{2}$) are essential for designing filter devices. Using AlN/AlScN composite film with the adjustable thickness ratio can be a feasible approach to obtain the required $K_{eff}^{2}$. In this work, we research the resonant characteristics of FBARs based on AlN/AlScN composite films with different thickness ratios by finite element method and fabricate FBAR devices in a micro-electromechanical systems process. Benefiting from the large piezoelectric constants, with a 1 μm-thick Al_0.8_Sc_0.2_N film, $K_{eff}^{2}$ can be twice compared with that of FBAR based on pure AlN films. For the composite films with different thickness ratios, $K_{eff}^{2}$ can be adjusted in a relatively wide range. In this case, a filter with the specific N77 sub-band is demonstrated using AlN/Al_0.8_Sc_0.2_N composite film, which verifies the enormous potential for AlN/AlScN composite film in design filters.

## 1. Introduction

For achieving a high-speed and large-capacity data exchange in the wireless communication, filters as key elements in the radio-frequency front-end module are desired to possess large bandwidth, high frequency, and low insertion [1,2,3,4]. Adopting aluminum nitride (AlN)-based film bulk acoustic resonators (FBARs) to construct filters are a promising approach to meet these requirements due to the high acoustic velocity of AlN, achievable large effective electromechanical coupling coefficient ($K_{eff}^{2}$) of FBAR and complementary metal oxide semiconductor compatibility [5,6]. B.P. Sorokin et al. have recently obtained an excitation of longitudinal bulk acoustic waves in a diamond-based high overtone bulk acoustic resonator at microwave and enhanced frequency bands up to 40 GHz [7,8]. However, with the rapid development of fifth-generation communication, the characteristics of AlN-based FBAR are further expected to be improved. For the design of the film bulk acoustic filters, $K_{eff}^{2}$ is a crucial parameter that affects the bandwidth and cutoff frequency of filters. In particular, doping method is an effective option to increase the electromechanical coupling coefficient ($K_{t}^{2}$) of piezoelectric material AlN, for example using Sc doping, thus obtaining an expected large bandwidth for AlN-based filters. Milena Moreira et al. have proved that using Sc doping with concentration of 15 at.% can achieve a two-times increase in the $K_{eff}^{2}$, which is suitable for the applications needing broad bandwidth [9].

However, for specific requirements of bandwidth and frequency, we may need suitable $K_{eff}^{2}$ for FBARs in order to achieve the accurate control in passband of filters. Although different Sc doping concentrations in AlN can effectively obtain a different value of $K_{eff}^{2}$ for AlN-based FBARs, the Sc alloy targets for sputter technology are costly and it is difficult to produce arbitrary concentrations. AlN/AlScN bilayer composite film is a potential choice to realize the modulation of $K_{eff}^{2}$ for FBARs, since we can modify the effective piezoelectric constants of the composite film using varying thickness ratios of AlN to AlScN films. In our previous work, AlN/AlScN bilayer composite film was selected to acquire a comparatively higher $K_{eff}^{2}$ of 7.8% for the Lamé Mode resonator [10]. Li et al. have a detailed investigation about the effective properties of AlN/AlScN bilayer composite film based on the Reuss model and Eshelby–Mori–Tanaka micromechanics theory and built an explicit relationship between piezoelectric constant *d*_33_ and the thickness ratio of AlN to AlScN [11]. Su et al., also found that AlN as a seed layer can effectively enhance the crystal quality and (002) orientation of AlScN film, which can be adopted to further improve the properties of FBAR and filter devices [12]. 

In this paper, we demonstrate the modulation of $K_{eff}^{2}$ for FBARs using different piezoelectric materials and propose the filter designs for specific bandpass based on the AlN/AlScN bilayer composite film. We investigate the influence of different thickness ratios of AlN to AlScN layer on the resonant characteristics and $K_{eff}^{2}$ of FBARs via finite element simulation. FBARs based on pure AlN, AlN/Al_0.9_Sc_0.1_N, and AlN/Al_0.8_Sc_0.2_N bilayer composite film are fabricated and we can obtain varying $K_{eff}^{2}$ for FBARs consistent with the simulated results. AlScN can effectively compensate the deficiency of AlN film in electromechanical coupling in the form of AlN/AlScN composite film. With a decreased thickness ratio of AlN to AlScN, an obvious increased $K_{eff}^{2}$ for FBARs can be realized. It is also verified that with AlN/Al_0.8_Sc_0.2_N composite film the filter for N77 sub-band (3.4 GHz–3.6 GHz) can be easily demonstrated, proving the feasibility using composite film to achieve the expected $K_{eff}^{2}$ for filter design. 

## 2. Materials and Methods

In our work, the resonant characteristics of FBARs based on AlN and AlN/AlScN bilayer composite film were simulated using finite element method. All the piezoelectric materials were deposited by a magnet sputter (SPTS, Sigma fxP system, Newport, UK) under 200 °C. Pure Al metal, Al-Sc alloys with the atomic mass percent of Sc of 10% and 20%, respectively, were adopted when depositing the piezoelectric films [13]. Sputter power of 6 kW and bias power of 160 W were used for the film deposition with the flow rates of N_2_ and Ar of 60 sccm and 20 sccm, respectively. X-ray diffraction (XRD) measurement (Rigaku, SmartLab SE with a Cu Kα radiation, Tokyo, Japan) was used to characterize the crystal structure of piezoelectric films [13]. 

**Figure 1:**
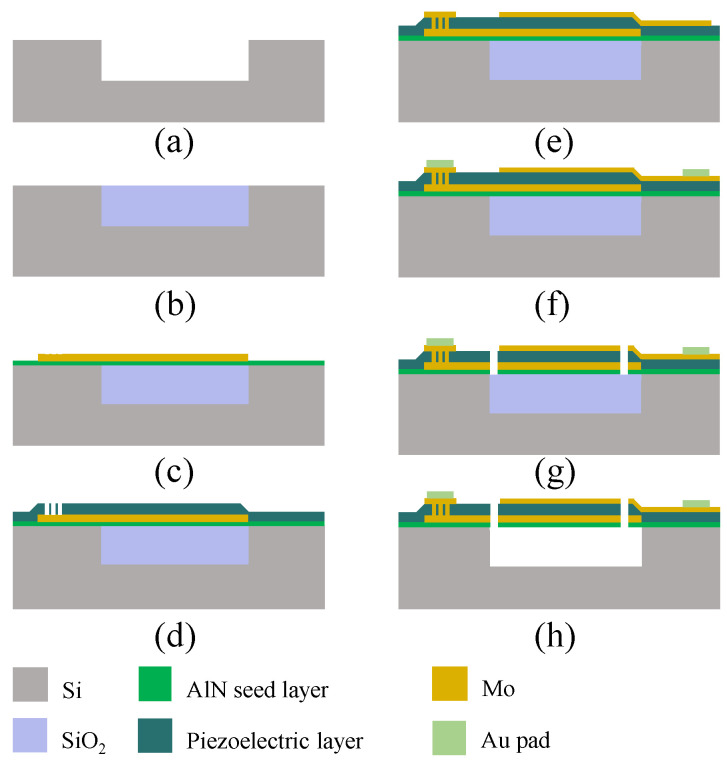
Main fabrication process steps of FBAR devices. (**a**) Etching Si substrate to form the cavity. (**b**) SiO_2_ sacrificial layer deposition and polished. (**c**) AlN seed layer and bottom Mo electrode deposition. (**d**) Piezoelectric layer deposition and etched. (**e**) Top Mo electrode deposition and patterned. (**f**) Au pad layer deposition. (**g**) Release windows opened. (**h**) Releasing SiO_2_ sacrificial layer to form the cavity.

FBAR devices based on the micro-electromechanical systems process were fabricated on 725 μm-thick silicon substrates and the impedance curves of FBAR devices were measured using Keysight network analyzer (Keysight, N5222B, Santa Rosa, CA, USA) connecting to a Cascade Microtech’s GSG probe station (FormFactor, Beaverton, OR, USA) [10]. The fabrication process flow is shown in Figure 1 [14]. The fabrication process started with etching Si to form the cavity (Figure 1a). The SiO_2_ was deposited by physical vapor deposition as the sacrificial layer and chemical mechanical polishing was used to polish the surface of SiO_2_ layer (Figure 1b) for the deposition of subsequent films. Then, AlN seed layer with a thickness of 25 nm and bottom Mo electrode layer were deposited using magnetron sputtering as shown in Figure 1c. Next, the piezoelectric layer (AlN, AlN/Al_0.9_Sc_0.1_N and AlN/Al_0.8_Sc_0.2_N composite films) was deposited by the magnet sputter and etched by inductively coupled plasma to open the bottom electrode pad as shown in Figure 1d. Another Mo layer was deposited and patterned as the top electrode layer (Figure 1e). Subsequently, the Au layer was deposited by electron beam evaporation and patterned (Figure 1f). The release windows were opened to release SiO_2_ in the cavity (Figure 1g). Finally, the SiO_2_ layer was wet-etched by HF/NH_3_F mixed solution to empty the cavity and the resonators were fabricated completely (Figure 1h). 

## 3. Results and Discussions 

**Figure 2:**
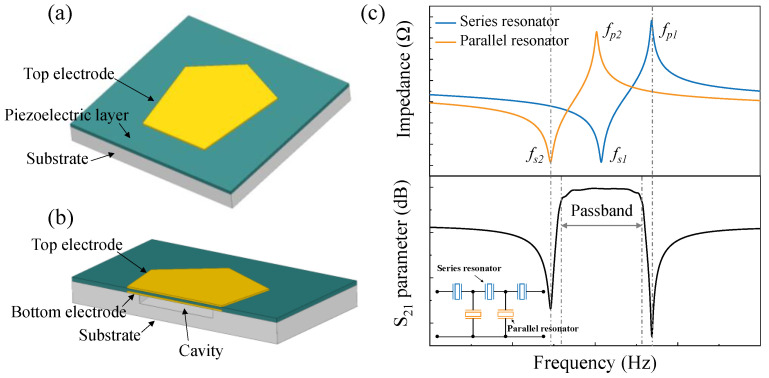
Structures of FBAR and characteristics of filters. (**a**) Schematic drawing of a typical FBAR. (**b**) The cross-sectional view of FBAR. (**c**) Working principle of filter based on FBARs. Inset shows the ladder circuit topology of filters.

Figure 2a,b show the schematic structures of a typical FBAR, which consists of a piezoelectric layer sandwiched between the top and bottom electrodes. The voltage or the electrical field between the two electrodes excites the acoustic wave. An air cavity is created between the bottom electrode and the substrate to trap the acoustic wave between the electrodes, as shown in Figure 2b. Figure 2c shows the working principle of a ladder filter based on FBAR, the inset in Figure 2c is the circuit topology of the ladder filters. The resonator has two resonant frequencies, one is the series resonant frequency *f_s_*, at which the impedance *Z_min_* can be very low, and the second one is a parallel resonant or anti-resonant frequency *f_p_*, at which the impedance *Z_max_* can be very high. The parallel resonator in the filter is tuned to be a slightly lower frequency by adding a mass loading layer on the top electrode. When *f_p2_* representing the anti-resonant frequency of parallel resonators is chosen to be equal to or slightly lower than *f_s1_* representing the series resonant frequency of series resonators, a passband is formed between the frequencies near *f*_*s2*_and *f_p1_*. The bandwidth of the filter is mainly determined by the effective coupling coefficient $K_{eff}^{2}$ of FBARs, which can be calculated by Equation (1). Therefore, for the filter design with a specific requirement in the passband, we need to consider the resonant frequencies of FBAR and seek a suitable $K_{eff}^{2}$ carefully [15,16,17].

(1) $$\begin{array}{c} K_{eff}^{2}=\frac{\pi^{2}}{4}\frac{f_{s}}{f_{p}}\frac{f_{p}-f_{s}}{f_{p}} \end{array}$$

**Figure 3:**
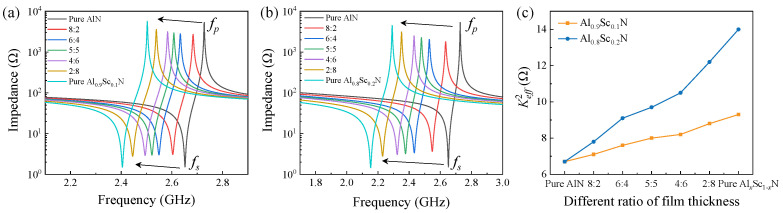
Simulated results of FBAR with different piezoelectric materials. (**a**) Simulated impedance curves of FBARs based on AlN/Al_0.9_Sc_0.1_N composite film with different thickness ratios. (**b**) Simulated impedance curves of FBARs based on AlN/Al_0.8_Sc_0.2_N composite film with different thickness ratios. (**c**) Calculated $K_{eff}^{2}$ of different FBARs.

For investigating the resonant characteristics of FBAR based on AlN/AlScN bilayer composite film with different thickness ratios, we used the finite element model to simulate the performances. Table 1 lists the material constants of AlN, Al_0.9_Sc_0.1_N, and Al_0.8_Sc_0.2_N piezoelectric films used for the simulation [11,18,19,20,21]. As shown in Figure 3, we obtained the impedance curves of FBARs with the pure AlN, AlN/Al_0.9_Sc_0.1_N, and AlN/Al_0.8_Sc_0.2_N composite films, respectively. The total thickness for piezoelectric layers is 1 μm, the thickness for both top and bottom Mo electrode is 200 nm. For FBAR based on 1 μm-thick AlN film, *f_s_* is 2.65 GHz and *f_p_* is 2.72 GHz. As shown in Figure 3a and Figure 3b, for FBAR with AlN/AlScN composite films, with the increased thickness ratio of AlScN to AlN, the resonant frequency decreases, which can be contributed to the lower longitudinal acoustic velocity of AlScN compared with the acoustic velocity of AlN [4,22,23]. Figure 3c shows $K_{eff}^{2}$ of FBARs with different piezoelectric materials calculated by Equation (1). When using AlN/AlScN composite film to replace pure AlN film, we can obtain an increased $K_{eff}^{2}$, and $K_{eff}^{2}$ for FBAR based on 1 μm-thick Al_0.8_Sc_0.2_N film can be twice of that when FBAR based on 1 μm-thick AlN film. It is also clear that using AlN/AlScN composite film with different thickness ratio can achieve an effective adjustment in $K_{eff}^{2}$, which can be adopted when designing filters with the expected requirement in passband [24,25]. 

**Figure 4:**
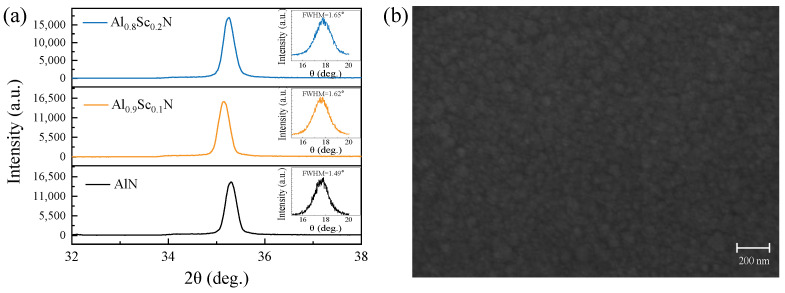
XRD results and morphology of piezoelectric materials. (**a**) XRD results of 1 μm-thick AlN, Al_0.9_Sc_0.1_N, and Al_0.8_Sc_0.2_N films, respectively. (**b**) Morphology of 1 μm-thick Al_0.8_Sc_0.2_N film.

We also deposited the piezoelectric films and fabricated FBARs to verify the simulated results. The pure AlN, Al_0.9_Sc_0.1_N, and Al_0.8_Sc_0.2_N with a thickness of 1 μm, respectively, were deposited on Si (100) substrate first. Further characterizations of piezoelectric materials were carried out using XRD as shown in Figure 4a; it is used to assess the (002) preferred orientation and crystal quality of piezoelectric films. Significant reflection peaks at around 35° to 36.0° associated with the (002) hexagonal AlN and AlScN films in patterns indicate that the piezoelectric films are well-crystallized with the *c* axis. The peak positions of Al_0.9_Sc_0.1_N and Al_0.8_Sc_0.2_N films shift due to the Sc doping [13,26,27]. The results of XRD rocking curves in the insets of Figure 4a show full width at half maximum (FWHM) of 1.49°, 1.62°, and 1.65° for 1 μm-thick AlN, Al_0.9_Sc_0.1_N, and Al_0.8_Sc_0.2_N films, respectively, suggesting a preferred c-axis crystal orientation as well [10,13]. Figure 4b shows the morphology of 1 μm-thick Al_0.8_Sc_0.2_N film caught by Scanning Electron Microscopy (SEM, Tescan, MIRA3, Brno, The Czech republic). Although small grain growth precipitates can be observed in the relative smooth surface of Al_0.8_Sc_0.2_N film, distinct clusters of particles, normally deteriorating the film quality, are absent [28,29]. 

**Figure 5:**
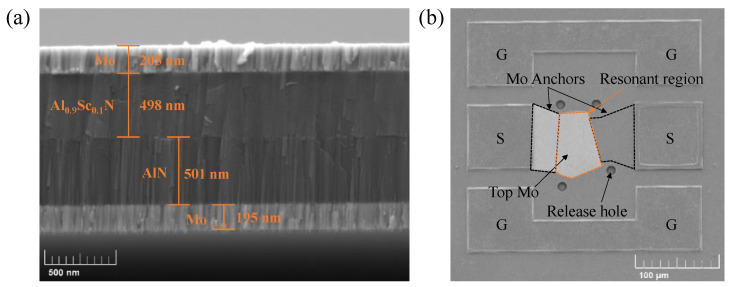
Characterization of piezoelectric film and FBAR device. (**a**) Cross-sectional view of AlN/Al_0.9_Sc_0.1_N composite film consisting of 500 nm-thick AlN and 500 nm-thick Al_0.8_Sc_0.2_N. (**b**) Vertical view of fabricated FBAR based on the AlN/Al_0.9_Sc_0.1_N composite film consisting of 500 nm-thick AlN and 500 nm-thick Al_0.8_Sc_0.2_N. The signal and ground pads are marked with “G” and “S” labels, respectively.

In our work, for the fabricated FBARs, three different piezoelectric layers were deposited, including 1 μm-thick pure AlN, 1 μm-thick composite piezoelectric layer comprising 500 nm-thick AlN and 500 nm-thick Al_0.9_Sc_0.1_N, and 1 μm-thick composite piezoelectric layer comprising 500 nm-thick AlN and 500 nm-thick Al_0.8_Sc_0.2_N. Figure 5a shows the cross-sectional view of FBAR based on 1 μm-thick AlN/Al_0.9_Sc_0.1_N composite film, in which the deposited thicknesses of AlN and Al_0.9_Sc_0.1_N layers are almost 500 nm, respectively, meaning we can achieve a delicate control for the film deposition. It can be seen that the films without obvious defects exhibit good flatness and crystal quality, which is essential for the performance of the device [27]. The vertical view of fabricated FBAR is shown in Figure 5b. It clearly shows that the resonant region is connected with signal pads via Mo anchors. The signal terminals on both sides of the resonator mean the input and output of electrical signals. Four release holes arranged at the corners of the edge are intended to etch the sacrificial layer fully and fabricate a resonant cavity.

**Figure 6:**
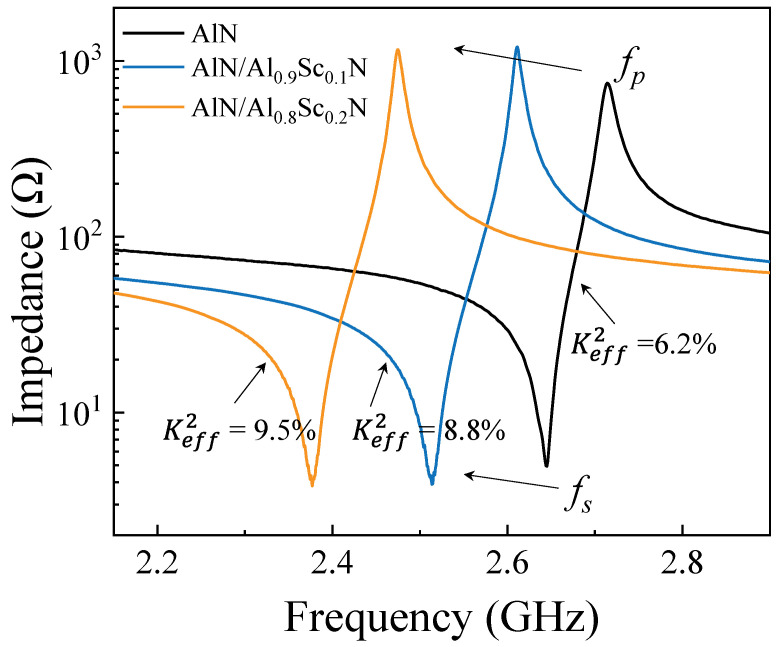
Tested impedance curves of FBARs with different piezoelectric materials: 1 μm-thick pure AlN, 1 μm-thick AlN/Al_0.9_Sc_0.1_N composite piezoelectric layer comprising 500 nm-thick AlN and 500 nm-thick Al_0.9_Sc_0.1_N, and 1 μm-thick AlN/Al_0.8_Sc_0.2_N composite piezoelectric layer comprising 500 nm-thick AlN and 500 nm-thick Al_0.8_Sc_0.2_N.

**Figure 7:**
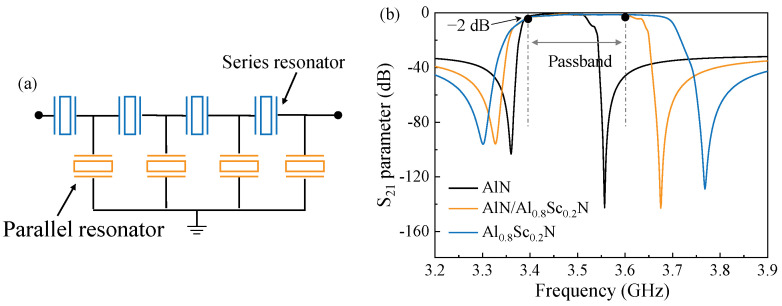
The proposed filter designs. (**a**) Schematic ladder circuit of the filter. (**b**) The comparison of simulated transmission responses among the filter 1 with AlN film, filter 2 with AlN/Al_0.8_Sc_0.2_N film, and filter 3 with Al_0.8_Sc_0.2_N film, respectively.

Figure 6 shows the tested impedance curves of FBARs based on pure AlN, AlN/Al_0.9_Sc_0.1_N, and AlN/Al_0.8_Sc_0.2_N composite film, respectively. The measured resonant frequencies and calculated $K_{eff}^{2}$ are closed with the simulated ones shown in Figure 3, which means that the estimated parameters in FBARs, including material constants, the thickness of each layer, are under control. Therefore, we can use the simulated conditions and results to evaluate the filter design. In order verify the feasibility of composite films for filter design with specific bandwidth, we take the design of N77 sub band (3.4 GHz to 3.6 GHz) for example. Here, we adopt three piezoelectric films, including pure AlN film, AlN/Al_0.8_Sc_0.2_N composite film, and pure Al_0.8_Sc_0.2_N film, to design the filter. Figure 7a shows the schematic circuit of the designed filters. It consists of eight elements, including four series and four parallel resonators. Table 2 summarizes the thickness information of these three filters. The simulated results of the filters are plotted in Figure 7b. We can find that filter 1 with pure AlN film cannot meet the demand of 200 MHz bandwidth due to the limited intrinsic electromechanical coupling factor. As for filter 3 with pure Al_0.8_Sc_0.2_N film, it demonstrates a bandwidth larger than 200 MHz. Remarkably, by combining the characteristics of AlN and Al_0.8_Sc_0.2_N films, the proposed filter 2 can well meet the bandwidth requirement of 200 MHz demonstrating the serviceability using AlN/AlScN composite film with different thickness ratios for specific passband and frequency.

## 4. Conclusions

In this work, we investigate the resonant characteristics of FBARs with different piezoelectric materials, including pure AlN, AlScN, and AlN/AlScN composite films, and explore the potential of using varying AlN/AlScN composite film to meet the requirements of filters with the expected bandwidth. We use finite element method to simulate the influence of different thickness ratio of AlN/AlScN composite film on the key parameter, $K_{eff}^{2}$, and have a detailed insight in the resonant characteristics by fabricating FBARs based on AlN, AlN/Al_0.9_Sc_0.1_N, and AlN/Al_0.8_Sc_0.2_N films. The results show that $K_{eff}^{2}$ can be increased by two times for FBAR based on 1 μm-thick Al_0.8_Sc_0.2_N film compared with that of FBAR based on 1 μm-thick pure AlN film. Adopting AlN/AlScN composite film with the adjustment in thickness ratio, we can achieve the delicate control on $K_{eff}^{2}$, which can be an effective method for the further filter design. This work paves a way for filter demonstration using AlN/AlScN composite film with varying $K_{eff}^{2}$ to achieve the specific passband and frequency.

## Figures and Tables

**Table 1:** Material constants of piezoelectric film used in the simulations.

	AlN	Al_0.9_Sc_0.1_N	Al_0.8_Sc_0.2_N
**ρ (kg/m^2^)**	3260	3460	3560
**ε_r_**	9.5	10.8	13.4
**e_31_ (C/m^2^)**	−0.58	−0.62	−0.71
**e_33_ (C/m^2^)**	1.55	1.67	2.08
**e_24_ (C/m^2^)**	−0.48	−0.30	−0.27
**C_11_ (GPa)**	345	320	292
**C_12_ (GPa)**	125	127	130
**C_13_ (GPa)**	120	126	134
**C_33_ (GPa)**	395	324	258
**C_44_ (GPa)**	118	108	104
**C_66_ (GPa)**	110	110	91

**Table 2:** Structure parameters of the designed filters.

Dimension	Filter 1	Filter 2	Filter 3
Thickness of bottom Mo(nm)	184	103	102
Thickness of AlN (nm)	686	-	316
Thickness of Al_0.8_Sc_0.2_N (nm)	-	679	473
Thickness of top Mo (nm)	164	103	100
Thickness of Mass loading Mo (nm)	21	41	30

## Data Availability

Data and code are available from the corresponding authors upon reasonable request.
